# Promoting HPV Vaccination in College Students Through Educational Video: Results from a Randomized Trial

**DOI:** 10.3390/vaccines13060587

**Published:** 2025-05-30

**Authors:** Angela Chia-Chen Chen, Kimberly Arcoleo, Alli Walsh

**Affiliations:** College of Nursing, Michigan State University, 1355 Bogue St, East Lansing, MI 48824, USA; arcoleok@msu.edu (K.A.); walshall@msu.edu (A.W.)

**Keywords:** college students, HPV vaccination, vaccination intention, video-based intervention

## Abstract

Objectives: Human papillomavirus (HPV) is a leading cause of several cancers, yet HPV vaccination rates among U.S. young adults remain low. This study evaluated the effect of a brief educational video, co-developed with college students, in increasing HPV vaccination intention among unvaccinated college-aged individuals. Methods: A two-group randomized controlled trial was conducted among 215 college students aged 18–26 who had not received the HPV vaccine. Participants were randomly assigned to two interventions: a video group (*n* = 111) or a leaflet group that viewed a CDC-based educational sheet (*n* = 104). Pre- and post-intervention surveys assessed HPV knowledge, perceived risk, facilitators and barriers to vaccination, cultural beliefs, and vaccination intention. Data were analyzed using descriptive statistics, *t*-tests, Wilcoxon Signed-Rank, and Mann–Whitney U tests. Results: At baseline, approximately 56% of the sample (*n* = 215; mean age = 23.5, 71.2% male) have learned about HPV in the past. Although both groups improved in HPV knowledge, perceived lower risk, and cultural beliefs, between-group differences in these theoretical mediators were not statistically significant. Vaccination intention (those who responded “Yes” or “Maybe”) increased by 10.8% in the video group but decreased by 11.6% in the leaflet group following the intervention; the difference between the two groups was statistically significant (*p* = 0.03). Conclusions: A brief, participatory, and theory-based video significantly increased HPV vaccination intent among unvaccinated college students. Compared to the leaflet intervention, the video intervention offers a promising and scalable public health strategy for promoting HPV vaccination in this high-risk population.

## 1. Introduction

Human papilloma virus (HPV), the most common sexually transmitted infection worldwide, is responsible for approximately 37,800 new cases of cancers annually in the United States. These include anal, cervical, oropharyngeal, penile, vaginal, and vulvar cancers affecting males and females [1]. Significantly, the annual direct medical cost of HPV-associated diseases in the United States was estimated to be approximately USD 9.01 billion over the period 2014–2018 (in 2020 U.S. dollars), with 43.8% attributed to the treatment of HPV-attributable cancers [2]. Of the 14 million new HPV infections occurring each year, almost half occur in youth and young adults aged 15–24 years old [3].

College students represent a high-risk group for HPV infection due to their age and sexual behavior patterns; however, they often exhibit lower levels of knowledge and awareness compared to other demographic groups. Research has identified significant knowledge gaps among college students regarding HPV transmission, associated health risks for both men and women, vaccine recommendations, and accessibility [4,5]. These gaps are especially concerning when contrasted with older, college-educated adults, who are more likely to have had exposure to preventive health messaging and consistent healthcare interactions. Although HPV vaccination efforts primarily target adolescents before college entry, many college students report having missed these opportunities during adolescence and begin college with limited HPV-related education [5]. Furthermore, a systematic review found the highest prevalence of HPV among female college students and women attending sexually transmitted disease clinics [6]. Collectively, these findings underscore the need for tailored, developmentally appropriate interventions that address the specific informational needs and health behaviors of college student populations. In the U.S., the HPV vaccine provides protection against six cancers and genital warts in males and females. Universal vaccinations are recommended for both females and males at age 11 or 12 [7]. Despite that the HPV vaccine has the potential to prevent more than 90% of HPV-related cancers, only 21.5% of U.S. young adults have completed the recommended doses [8].

Lack of HPV-related knowledge, including limited awareness of eligibility criteria and vaccine availability, has been identified as a key factor contributing to low rates of HPV vaccination among both male and female college students [9,10]. Notably, one of the strongest predictors of HPV vaccination in this population is having heard about the vaccine [11]. Additional barriers include perceived low risk of getting infected, misperceptions about HPV infection (e.g., HPV primarily affects women or is only linked to cervical cancer) and vaccine, concerns about vaccine safety and side effects, stigma associated with HPV and other sexually transmission infections, and absence of recommendation from parents and peers [9,10]. Low rates of routine healthcare utilization among college students, along with missed opportunities by healthcare providers to discuss or recommend the HPV vaccine, and perceived barriers such as cost and lack of insurance coverage, further contribute to suboptimal uptake [9,10,12]. Importantly, research indicates that the provision of general information on sexually transmitted infections and discussions with healthcare providers are positively associated with HPV vaccination [10,12].

Multiple sociocultural factors also influence HPV vaccination rates among college-aged adults. For example, residing or attending college in the southern United States has been associated with lower vaccination uptake [12]. Additionally, disparities exist across racial and ethnic groups. According to Thompson et al. [12], women from ethnic minority backgrounds were significantly less likely to report receiving the HPV vaccine compared to their male counterparts; for example, African American women were approximately 40% less likely to be vaccinated than African American men. These findings underscore the complex interplay of sex, race, and access that contributes to uneven vaccination rates among college students. Among young adults, parental attitudes and influence remain significant determinants of vaccination behavior [9]. These patterns are often shaped by prevailing social and religious norms, some of which conflict with the promotion and acceptance of the HPV vaccine [9].

Based on a systematic review, educational interventions aimed at promoting HPV vaccination are effective in increasing both knowledge and vaccination intentions among college students [13]. Importantly, vaccination intention was positively associated with higher rates of vaccine initiation and completion in this population. Effective interventions were largely theory-driven and delivered through a variety of formats, including web-based platforms, text messaging, and video content. Among these, educational videos were found to be particularly effective, outperforming other formats such as printed materials or usual care in enhancing knowledge, and influencing vaccine acceptance and uptake. For example, Valdez et al. [14] reported an 81.6% improvement in knowledge in a group shown a video, compared to 51.1% in a group shown a CDC flyer, while Krawczyk et al. [15] observed a 45.3% increase in a group shown a video, compared to 10.7% in a control group. Regarding vaccine willingness or acceptability, Cory et al. [16] found that 51.7% of participants in a group shown a video expressed willingness to vaccinate, compared to 33.3% in a group given a handout and 28.2% in a control group. Similarly, Valdez et al. [14] reported a 21.3% higher acceptability rate in a group shown a video, compared to a group shown a CDC flyer. In terms of vaccine uptake, Hopfer [17] reported a 10% higher initiation rate in a group shown a video compared to a control group, and Vanderpool et al. [18] found that participants who received a video intervention alongside usual care were 2.44 times more likely to complete the HPV vaccine series than those who received usual care alone. Furthermore, consistent with findings from the systematic review, interventions grounded in behavioral theories and those incorporating cultural tailoring have been shown to foster more favorable attitudes toward HPV vaccination and greater knowledge acquisition [18,19,20,21].

The Health Belief Model (HBM) is one of the most widely used frameworks for understanding HPV vaccination decisions [22]. Its key constructs such as perceived risk (belief about the risk of contracting a health issue), perceived benefits and barriers (beliefs about the effectiveness of and obstacles to health actions), and perceived facilitators or cues to action (external prompts such as healthcare provider’s recommendations) have shown strong associations with HPV vaccination intentions and behaviors [23]. The HBM also incorporates sociodemographic and structural factors such as sex, gender, and race/ethnicity that can shape individuals’ perceptions of health risks and benefits. Measures assessing these theoretical constructs were included in this study to understand their relationships with vaccination intention.

Although there is a growing attention to HPV prevention among college students, several research gaps remain in understanding and improving their vaccination rates. Much of the existing literature has focused on females, despite the importance of vaccinating both sexes for cancer prevention. Existing interventions often overlook the unique social, developmental, and behavioral contexts of college students, such as their transitional health autonomy, low perceived risk of infection, and limited engagement with preventive care. To maximize effectiveness, video content must go beyond generic messaging and address the specific informational needs, cultural diversity, and communication preferences of college students.

Engaging key stakeholders, such as college students, in the co-development of video interventions ensures that content is accurate, culturally and developmentally relatable, and reflective of real campus experiences. This participatory approach enhances message credibility and resonance, which are crucial for influencing attitudes and behaviors [24,25,26]. Furthermore, videos co-created with college students are more likely to incorporate authentic language, peer perspectives, and preferred media platforms, factors that significantly improve engagement and vaccine uptake. Addressing these gaps presents a valuable opportunity to create impactful, scalable interventions that align with the needs and realities of today’s college student populations.

### Study Aim

Using a participatory approach, we co-developed an educational video with college students to address low HPV vaccination rates within this population. This randomization trial aimed to examine the effect of a video intervention on vaccination intention among college students aged 18–26, compared to the provision of a leaflet intervention consisting of a CDC-based educational sheet.

## 2. Materials and Methods

### 2.1. Study Design

This study consisted of two phases. Phase I focused on co-designing an educational video with college students, while Phase II involved a randomized controlled trial (RCT) to examine the effects of two interventions (video and leaflet) on HPV vaccination intention among unvaccinated college students aged 18–26.

Phase I: Video Co-Design Process. The educational video was co-developed through a participatory process involving college students and faculty. Three undergraduate students with varied HPV vaccination statuses collaborated with faculty to create the script, visuals, and messaging tone, ensuring cultural and developmental relevance, informational accuracy, and alignment with behavioral theory. This collaborative approach produced a final product that authentically reflected students’ lived experiences, communication preferences, and campus realities, thereby enhancing the video’s credibility, relatability, and engagement potential among the target audience. The students who participated in the co-design process were excluded from Phase II.

Phase II: RCT. We conducted a two-arm RCT to evaluate the effect of two different interventions among college students aged 18–26 who had not yet received HPV vaccine.

### 2.2. Sample and Sampling

A convenience sample of 215 students aged 18–26 was recruited from Amazon Mechanical Turk (MTurk). Inclusion criteria: participant who (a) has not received HPV vaccines; (b) reads and understands English; (c) lives in the United States; (d) between the ages of 18 and 26; (e) submits the consent online.

### 2.3. Procedures

Following institutional review board approval, interested individuals completed a brief online screening form to confirm eligibility. Eligible participants were directed to a webpage outlining the study’s purpose, procedures, potential risks and benefits, and the consent form. Upon consenting, they completed the pre-intervention survey. One week later, participants were randomly assigned to one of two study conditions: a brief video intervention or a leaflet intervention featuring a CDC-based educational sheet. After viewing the assigned material, participants immediately completed the post-survey. Each survey took approximately 10–15 min, and the intervention material required about 3–5 min. Participants received a total of USD 6.50 for completing the baseline survey (USD 1.50) and the post-intervention survey (USD 5.00), consistent with MTurk compensation norms of approximately USD 1.00 per 10 min of work. Data were saved and managed using REDCap and then imported to SPSS (Version 28.0) for analysis.

We recruited participants via MTurk, a widely used crowdsourcing platform that offers a cost-effective and efficient means of reaching diverse adult populations. MTurk has demonstrated advantages in participant attentiveness and compliance compared to other convenience sampling methods [27], as well as for the relative validity and reliability of data it yields in health and medical research contexts [28,29]. While prior research has noted that MTurk samples often include younger, non-minority individuals with higher educational attainment relative to nationally representative datasets [30,31,32], evidence has also shown that it is possible to successfully recruit racial/ethnic minorities using this platform [33,34]. To enhance our ability to reach a diverse sample of college students, we utilized a multi-stage survey design strategy as recommended in practice-based crowdsourcing literature [35]. Two attention-check questions were embedded in the survey to assess participant attentiveness and ensure data quality, consistent with prior research targeting college students [36]. All participants passed attention checks; thus, no exclusions were made for inattentiveness.

### 2.4. Measures

Participants completed a pre- and a post-test survey measuring multiple factors relevant to HPV vaccination, including sociodemographic characteristics, HPV knowledge, perceived risk of being infected, facilitators and barriers of vaccination, and vaccination intent. The measures included in the survey were drawn from empirical evidence and theoretical constructs known to be associated with behavioral intention and changes. All survey questions were pilot-tested in prior research and found to be reliable and valid [37,38,39,40,41].

Sociodemographics: sociodemographic characteristics such as age, race/ethnicity, sex, marital status, college enrollment, and academic major were collected.

HPV Knowledge. A 10-item true/false scale, developed based on CDC recommendations [42], was used to assess participants’ knowledge of HPV. Items covered topics such as differences in male versus female risk and symptoms, HPV vaccination, and related health risks (e.g., “Both males and females can be infected with HPV”). Correct responses were coded as 1 and incorrect responses as 0. Total knowledge scores were calculated by summing correct responses (range: 1–10). Higher scores indicated greater HPV-related knowledge. Internal consistency reliability was modest (α = 0.60 at T0 and 0.62 at T1 in this sample), which is not uncommon for brief, dichotomously scored knowledge scales covering diverse content areas.

Perceived Facilitators. Twelve yes/no items assessed perceived facilitators of HPV vaccination, including beliefs about disease prevention, social influence, access, and affordability (e.g., “I want to do it to save my life”). Affirmative responses were coded as 1 and summed to create a total score (range: 1–12), with higher scores indicating stronger perceived facilitator influence. Internal consistency reliability was acceptable (α = 0.70 at T0 and 0.73 at T1).

Perceived Barriers. Sixteen yes/no items were used to assess perceived barriers to HPV vaccination, including concerns related to age, access, cost, vaccine safety, personal attitudes, and peer influence (e.g., “Because none of my friends get it”). Affirmative responses were coded as 1 and summed (range: 1–16), with higher scores indicating greater perceived barriers. Internal consistency was high (α = 0.86 at T0 and 0.87 at T1).

Perceived Low Risk of Contracting HPV. Perceptions of low personal risk for contracting HPV were assessed using nine true/false items (e.g., “No one in my family has HPV infection, so I won’t get it”). Higher scores reflected *lower* perceived risk. Scores ranged from 1 to 9, with strong internal consistency (α = 0.80 at T0 and 0.85 at T1).

Cultural Beliefs: cultural beliefs linked to the need for HPV vaccination. We adapted three items from the Chance Health Locus of Control subscale, which assesses the belief that health outcomes are determined by fate, luck, or destiny [43]. In this study, the items were tailored to capture beliefs reflecting the Chance Locus of Control specifically related to HPV vaccination. Responses were coded on a five-point Likert-type scale (1 = *strongly disagree* to 5 = *strongly agree*). Higher scores indicated stronger beliefs that HPV-related health outcomes are determined by fate, luck, or destiny, which lessen the perceived need for HPV vaccination. Scores ranged from 3 to 15, with high internal consistency (α = 0.89 at T0 and 0.90 at T1).

Vaccination Intention and Reasons: Intention to receive the HPV vaccine was measured using a single question with three response options: “No (1)”, “Maybe (2)”, or “Yes (3)”. Higher scores indicated stronger intention to receive the HPV vaccine. Participants also reported reasons for their vaccination intention response, with up to five points assigned to each response based on the number of reasons they endorsed (one point per reason).

### 2.5. Power and Data Analysis

An a priori power analysis using G*Power 3.1 indicated that a total sample size of 88 participants (44 per group) would be required to detect a medium effect size (w = 0.3) with 80% power at a 0.05 significance level using a Chi-square test of independence. Our actual sample size of 215 (*n* = 111 video, *n* = 104 leaflet) exceeds this requirement, demonstrating adequate power to identify intervention effects. Oversampling was intentional to increase statistical power for detecting small effect sizes, particularly given the short interval between the pre- and post-intervention surveys. Additionally, a larger sample size allowed us to ensure diversity in the sample based on the key demographics.

We conducted descriptive univariate analyses (means, frequencies, standard deviations) to characterize the sample and key variables. Independent and paired-samples *t*-tests were conducted to examine between-group (video vs. leaflet) and within-group (pre- and post-intervention) differences in theoretical mediators. The Mann–Whitney U test was used to assess the effect of the intervention on the ordinal outcome measure (vaccination intention) between the two groups, while the Wilcoxon Signed-Rank test was employed to evaluate within-group changes in vaccination intention over time.

## 3. Results

### 3.1. Baseline/Pre-Intervention Comparison of Sociodemographic Characteristics by Study Condition

The mean age was 23.51 years (SD = 2.39) for the whole sample (N = 215). The majority of participants were male (71.2%) and pursuing health-related majors (79.7%). Most participants (81.6%) reported having health insurance, and 56.3% had heard about HPV prior to the intervention. We compared these baseline measures between the two groups and the results indicated no significant differences in any of the variables (see Table 1).

### 3.2. Within- and Between-Group Comparisons of Theoretical Mediators by Study Condition over Time

We examined within-group and between-group differences in theoretical mediators (knowledge, perceived facilitators, perceived barriers, perceived risk of contracting HPV, and cultural beliefs) over time (Table 2). Pre-intervention comparisons between groups were conducted to confirm equivalence prior to the intervention and identify any potential covariates that would need to be accounted for in the analyses, ensuring internal validity despite the use of a non-representative convenience sample. Both groups demonstrated significant improvements in the expected directions for mean scores. Knowledge scores increased by 0.77 in the video group and 0.09 in the leaflet group; perceived risk scores improved by 1.07 and 0.71, respectively. Perceived barriers decreased by 1.14 and 0.88; and cultural beliefs scores improved by 1.61 and 1.10, respectively. Although perceived facilitators increased in both groups (0.01 vs. 0.07), these changes were not statistically significant. As shown in Table 2, no statistically significant between-group differences in theoretical mediators were observed at either baseline or post-intervention.

### 3.3. Within- and Between-Group Comparisons of Intervention Effect

Table 3 shows that within the *intervention* group who watched the educational video, the response “Yes” for vaccination intent increased from 9.9% to 16.2%, the response “Maybe” increased from 24.3% to 28.8%, and the response “No” dropped from 65.8% to 55.5% after the intervention (*p* < 0.001). Within the leaflet group who read the CDC HPV education sheet, the response “Yes” for vaccination intent remained at 14.4% over time, the response “Maybe” dropped from 26.0% to 14.4%, and the response “No” increased from 59.6% to 71.2% after the intervention (*p* < 0.001). The Mann–Whitney U test was used to compare HPV vaccination intention between the groups at both pre- and post-intervention (Table 3). No significant differences were observed between groups at pre-intervention; however, at post-intervention, the video group reported significantly higher vaccination intention than the leaflet group (*p* = 0.03).

Additionally, participants provided reasons for their vaccination decision following their responses about vaccination intention post-intervention. Among those who re-sponded ‘Yes’ to vaccination intention, the primary reasons cited were the following: ‘It’s safe (79.3%)’, ‘It will protect me from HPV-related cancers and diseases (61.5%)’, and ‘It will keep me healthy (48.9%)’. Students who selected ‘Maybe’ commonly expressed uncertainty, with reasons such as ‘I’m not sure if it will keep me healthy (44.7%)’, ‘I’m not sure if it’s safe (42.6%)’, and ‘I’m not sure if it will help prevent HPV-related cancers and diseases (38.3%)’. Those who responded ‘No’ indicated that ‘I don’t need it because I don’t see any risk of contracting HPV (30.3%)’, ‘I don’t trust vaccines (30.3)’, and ‘Vaccines are toxic (24.2%)’ as their main reasons.

## 4. Discussion

This study demonstrated that a brief educational video co-developed with college students can significantly increase HPV vaccination intent among unvaccinated college students aged 18–26. Our findings align with previous research indicating that brief, tailored video interventions are more effective than traditional printed materials in promoting HPV vaccination among young adults, including college students [16,17]. Further, the differences in knowledge, perceived risk, facilitators, barriers, and cultural beliefs between the two groups were in the desired direction although not statistically significant.

Importantly, the increase in vaccination intent among those who were initially uncertain (the “Maybe” responders) highlights a promising opportunity for public health efforts. Ayikoru et al. [44] underscore that vaccine acceptance exists on a continuum. Addressing the specific concerns of individuals, particularly those who are undecided, can be pivotal in shifting them toward greater acceptance. Undecided individuals may be more amenable to change, and tailored, engaging interventions like the video intervention used in this study may help shift them toward a vaccination decision. This finding aligns with the prior literature demonstrating that interventions are particularly effective in increasing vaccination intention among those who are not firmly opposed [16,17]. Among participants in the leaflet group, 12 shifted their intention from ‘Maybe’ to ‘No’, while there was no change among those who initially responded ‘Yes’. This shift may potentially be attributed to the leaflet’s formal and impersonal tone, lack of cultural relevance, and limited emotional engagement.

Vaccination hesitancy, however, remains a challenge. Participants in this study cited a lack of trust in the vaccine and perceived low personal risk (30.3% for each reason among participants who responded “No”) as primary reasons for opposing vaccination. These concerns mirror national trends and underscore the need for strategies that go beyond information provision. Addressing misinformation, increasing trust in science and healthcare systems, and emphasizing the relevance of HPV risk to all genders are critical components of future interventions [9,10]. The inclusion of culturally relevant beliefs, particularly those aligned with the external health locus of control (i.e., HPV-related health outcomes are determined by fate, luck, or destiny), adds depth to our understanding of HPV vaccine hesitancy. Participants in the video group reported stronger cultural beliefs related to the perceived need for vaccination, which may have influenced their vaccination intentions. Future research should explore how culturally tailored messaging can acknowledge these beliefs while reframing HPV vaccination as a proactive and culturally congruent health behavior among college students.

Interestingly, although the video group demonstrated higher vaccination intent, they also reported more perceived barriers compared to the leaflet group following the intervention. This paradoxical finding may be explained by the intervention increasing participants’ awareness of not only the benefits of vaccination, but also the potential challenges involved in getting the vaccine. Interventions, particularly those grounded in behavioral theories like the HBM, often raise awareness of both facilitators and barriers to action [22,23]. As participants become more informed, they may be better able to identify potential obstacles that they had not previously considered. The video intervention may have prompted more critical reflection on personal circumstances and external influences such as vaccine safety concerns, cost, access, or peer and cultural norms that could hinder the action [9,10], especially for those who were newly considering vaccination. This increased recognition of barriers does not necessarily indicate resistance; rather, it may reflect a deeper cognitive engagement with the decision-making process [19]. Evidence has shown that awareness of barriers can coexist with motivation to act, particularly when individuals perceive the benefits of vaccination to outweigh the obstacles [16,17]. It is also possible that the video intervention offered strategies to address these barriers, thereby enhancing participants’ intention to vaccinate. Future interventions may benefit from incorporating educational content with practical strategies to overcome common barriers, such as guidance on vaccine access on campus and locally, cost coverage, and strategies of initiating conversations with healthcare providers [14,34].

Notably, our sample overrepresented male (71.2%) and Asian (37.7%) students while underrepresenting female, White, and Black students, which differs from the national profile of U.S. college students; Hispanic and AI/AN students were proportionally represented [45]. The overrepresentation of male participants and students pursuing health-related majors likely reflects the characteristics of the Amazon Mechanical Turk (MTurk) user base and the self-selection nature of online survey participation. Prior research has shown that MTurk samples can vary demographically depending on survey topic and recruitment wording, with men being more likely to participate in studies involving health decision-making or vaccination [31]. Similarly, students in health-related majors may have been more inclined to engage with a study on HPV vaccination due to personal interest or perceived relevance, even though no specific recruitment targeting was employed. These demographic differences likely reflect the characteristics of MTurk-based recruitment and should be taken into account when interpreting the generalizability of our findings.

Overall, the findings support the potential of a theory-driven, culturally and developmentally appropriate educational video intervention co-designed with the key stakeholders to promote HPV vaccination among college students. The participatory approach, which involved the target population in content and format development, likely contributed to its effectiveness by enhancing its relatability, clarity, and credibility [24,26].

### Limitations

Several limitations should be considered when interpreting the findings of this study. First, the use of a convenience sample recruited through MTurk limits generalizability. Although MTurk can reach a diverse adult population, participants may not fully represent the broader population of college students in terms of sociodemographics, educational environments, or healthcare access. Specifically, the overrepresented male students, Asian students, and those in health-related majors, likely reflecting MTurk recruitment patterns and self-selection bias, thereby limit the generalizability of our findings to the broader U.S. college population. Second, although intention is an important proximal predictor of behavior, it does not always translate into action. The reliance on self-reported vaccination intention rather than actual vaccine uptake limits conclusions about long-term behavioral impact. Third, the study used a brief, single-session intervention and did not assess long-term retention or sustained changes in knowledge, attitude, or intention. Additionally, although validated measures were used, some scales such as knowledge had a relatively low internal consistency; further psychometric validation is warranted.

## 5. Conclusions

This study provides evidence that a brief, theory-based, culturally and developmentally relevant video co-developed with college students can significantly increase HPV vaccination intention among their unvaccinated peers. By engaging students in the design process, this participatory approach ensured that the video content reflected the language, concerns, cultural values, and lived experiences of the target population, which likely enhanced message credibility and emotional resonance. Involving students also helped tailor the format and tone to their communication preferences, making the content more engaging and accessible. The findings highlight the value of participatory, multimedia public health strategies to address vaccine hesitancy and promote preventive health behaviors in college populations. Given its brevity and scalability, the video could be integrated into college health campaigns, student orientation modules, or social media outreach to increase HPV vaccine awareness and uptake. Future research should explore ways to sustain and translate these intentions into actual vaccine uptake behaviors and address structural and cultural barriers that continue to shape vaccination behaviors.

## Figures and Tables

**Table 1:** Sample characteristics of participants by study condition.

Variables	All Participants (N = 215)		Video (N = 111)		Leaflet (N = 104)		Test Statistics ^b^	*p*
	Mean/SD	*n*/%	Mean/SD	*n*/%	Mean/SD	*n*/%		
Age ^a^	23.51 (2.39)		23.63 (2.42)		23.38 (2.37)		−0.25	0.453
Gender								
Male		153 (71.2)		82 (73.9)		71 (68.3)	1.98	0.373
Female		61 (28.4)		28 (25.2)		33 (31.7)		
Other		1 (0.5)		1 (0.9)		0 (0.00)		
Race/Ethnicity								
Non-His. White		78 (36.3)		40 (36.0)		38 (36.5)	1.54	0.820
Non-His. Black		7 (3.3)		4 (3.6)		3 (2.9)		
Non-His. AI/AN		4 (1.9)		1 (0.9)		3 (2.9)		
Non-His. Asian		81 (37.7)		41 (36.9)		40 (38.5)		
Hispanic/Latino		45 (20.9)		25 (22.5)		20 (19.2)		
Major								
Health-related		153 (79.7)		77 (79.4)		76 (80.0)	0.01	0.915
Not health-related		39 (20.3)		20 (20.6)		19 (20.0)		
Health insurance								
Yes		173 (81.6)		89 (17.6)		84 (80.8)	0.10	0.758
No		39 (18.4)		19 (82.4)		20 (19.2)		
Prior learning about HPV?								
Yes		121 (56.3)		59 (53.2)		62 (59.6)	0.91	0.340
No		94 (43.7)		52 (46.8)		42 (40.4)		

Note: SD = standard deviation; Non-Hispanic AI/AN = Non-Hispanic American Indian/Alaska Native. ^a^ Mean (SD) if variables are continuous, *n* (valid %) if variables were categorical. ^b^ t statistic for numeric variable and Chi-square statistic for categorical variable.

**Table 2:** Change in theoretical mediators pre- to post-intervention: video vs. leaflet.

Variables	Video (*n* = 111)			Leaflet (*n* = 104)			Intervention Effect (Comp-Int)	
	Mean (SD)	Δ (95% CI) ^a^	*p*-Value ^b^	Mean (SD)	Δ (95% CI) ^a^	*p*-Value ^b^	DiD (95% CI) ^b^	*p*-Value
Knowledge								
Pre-intervention	7.14 (1.92)			6.88 (1.87)			−0.27 (−0.78, 0.24)	0.30
Post-intervention	7.92 (1.35)	−0.77 (−1.16, −0.39)	<0.001	7.72 (1.46)	−0.09 (−1.24, −0.45)	<0.001	−0.20 (−0.58, 0.18)	0.30
Perceived Low Risks ^c^								
Pre-intervention	5.09 (2.83)			4.96 (2.69)			−0.14 (−0.88, 0.61)	0.72
Post-intervention	4.02 (3.03)	1.07 (0.55, 1.60)	<0.001	4.25 (2.99)	0.71 (0.26, 1.16)	0.002	0.23 (−0.58, 1.04)	0.58
Perceived Facilitators								
Pre-intervention	8.22 (2.38)			7.92 (2.32)			−0.29 (−0.93, 0.34)	0.36
Post-intervention	8.21 (2.54)	0.01 (−0.54, 0.56)	0.97	7.99 (2.40)	−0.07 (−0.59, 0.45)	0.80	−0.22 (−0.89, 0.45)	0.52
Perceived Barriers								
Pre-intervention	9.09 (4.50)			8.63 (4.58)			−0.49 (−1.71, 0.73)	0.43
Post-intervention	7.95 (4.71)	1.14 (0.28, 1.99)	0.009	7.73 (4.53)	0.88 (0.14, 1.63)	0.02	−0.21 (−1.46, 1.04)	0.74
Cultural Beliefs ^c^								
Pre-intervention	10.78 (3.40)			10.34 (3.44)			−0.45 (−1.37, 0.47)	0.34
Post-intervention	9.16 (3.94)	1.61 (0.93, 2.30)	<0.001	9.24 (3.86)	1.10 (0.45, 1.74)	0.001	0.08 (−0.98, 1.13)	0.89

Abbreviations: DiD, difference-in-differences; LS, least squares; obs, observations. ^a^ Within-group effect relative to baseline. ^b^ Between-group effects over time. ^c^ Higher scores represent a lower perceived risk of contracting HPV and stronger cultural beliefs associated with a diminished perceived need for HPV vaccination.

**Table 3:** Change in vaccination intention pre- to post-intervention: video vs. leaflet.

Variable	Video (*n* = 111)		Leaflet (*n* = 104)		Intervention Effect (Comp-Int)	
	N (%)	*p*-Value ^a^	N (%)	*p*-Value ^a^	DiD (*n*, %)	*p*-Value ^b^
Vaccination Intention-NO						
Pre-intervention	73 (65.8)		62 (59.6)		−11 (−6.2)	
Post-intervention	61 (55.0)		74 (71.2)		13 (16.2)	
Vaccination Intention-MAYBE						
Pre-intervention	27 (24.3)		27 (26.0)		9 (1.7)	
Post-intervention	32 (28.8)		15 (14.4)		−14 (−14.4)	
Vaccination Intention-YES						
Pre-intervention	11 (9.9)		15 (14.4)		4 (4.5)	
Post-intervention	18 (16.2)		15 (14.4)		−3 (−1.8)	
Overall Effect		<0.001		<0.001		0.03

Abbreviations: DiD, difference-in-differences; CI, confidence interval. ^a^ Within-group effect relative to baseline. ^b^ Between-group effects over time.

## Data Availability

Data from this study are available from the corresponding author upon reasonable request and with appropriate IRB approval.
